# In Vitro Antiatherothrombotic Effects of Extracts from* Berberis Vulgaris* L.,* Teucrium Polium* L., and* Orthosiphon Stamineus* Benth

**DOI:** 10.1155/2019/3245836

**Published:** 2019-03-14

**Authors:** Nurul Huda Mohd Nor, Fauziah Othman, Eusni Rahayu Mohd Tohit, Sabariah Md Noor, Rosniza Razali, Hazlina Ahmad Hassali, Hadijah Hassan

**Affiliations:** ^1^Department of Human Anatomy, Faculty of Medicine and Health Sciences, Universiti Putra Malaysia, Selangor, Malaysia; ^2^Department of Pathology, Faculty of Medicine and Health Sciences, Universiti Putra Malaysia, Selangor, Malaysia; ^3^Technology Pharmaceutical Group, Medical Technology Department, Malaysian Nuclear Agency, Kajang, Selangor, Malaysia; ^4^Food and Science Technology Research Centre, MARDI, Serdang, Selangor, Malaysia

## Abstract

Coronary artery disease is the leading cause of mortality and morbidity worldwide. The pathogenesis is mainly due to atherosclerosis, plaque rupture, and platelet thrombus formation. The main risk factors for coronary artery disease include obesity, hypercholesterolemia, smoking, diabetes, and high blood pressure. As a part of disease management, treatment options using anticoagulant and antiplatelet drugs can be applied with addition to lipid-lowering medication. However, medicinal plants comprising antiatherothrombotic effects can be used as options to combat the disease rather than drug therapies with lesser adverse effects. Therefore, the haematological effect of* Berberis vulgaris *L.*, Teucrium polium *L., and* Orthosiphon stamineus *Benth extracts was studied using in vitro model to prevent and to treat coronary atherothrombotic disease. The aqueous, methanol, and polysaccharide extracts of* B. vulgaris, T. polium,* and* O. stamineus,* respectively, were studied for their anticoagulant and antiplatelet effect on human whole blood. Extracts were subjected to the prothrombin time (PT) and activated partial thromboplastin time (APTT) test for anticoagulant activity. The antiplatelet activity was investigated using an electrical impedance method.* B. vulgaris* aqueous extract (BVAE),* B. vulgaris* polysaccharide extract (BVPE),* T. polium *aqueous extract (TPAE), and* T. polium *polysaccharide extract (TPPE) significantly prolonged the coagulation time in a concentration-dependent manner (*p*<0.05). The administration of BVAE demonstrated the most effective antiplatelet activity against platelet aggregation caused by arachidonic acid (AA) and collagen. These antiplatelet activities may correspond to the presence of higher total phenolic compound, which thus inhibit the platelet aggregation activity. In conclusion, these findings provide strong evidence on the antiatherothrombotic effect of BVAE and TPAE.

## 1. Introduction

The World Health Organization reported that coronary artery disease (CAD) is the leading cause of death and disability in the world [1]. Globally, it is estimated that about 17.3 million people died from CAD in 2008, and, by 2030, approximately 23.6 million of global mortality will be from this disease [2]. The risk factor for CADs is mainly due to atherosclerosis which leads to arterial thrombosis. 

The pathogenesis of atherothrombosis started with endothelial disturbance by many noxious stimuli, including oxidised LDL cholesterol, glycation end-products, smoking, and hypertension. Subsequently, the endothelial disturbance could lead to inflammation, oxidation of more lipoproteins, smooth muscle cell proliferation, platelet activation, hypercoagulation, and thrombosis formation. Hypercholesterolemia with high LDL increases the release of platelet-activating factor, which in turn increases the production of inflammatory factors including activation of macrophages and releasing of cytokines. The process encompasses chronic progressive atherosclerosis, punctuated by acute processes such as plaque rupture and platelet thrombus formation around progressive stenosis regions. The final pathway to arterial thrombosis is atherosclerosis plaque rupture. Thus, the effective way to treat or to prevent the process of thrombosis formation is by limiting or eradicating platelet-dependent thrombus formation.

The application of anticoagulant and antiplatelet drugs in addition to lipid-lowering medication is the cornerstone of coronary artery disease management. An ideal anticoagulant should be effective and safe, lack serious toxicity, and have a wide therapeutic window with minimal monitoring. It should also be available for oral long-term use, safe during pregnancy, having low cost and short half-life for drugs used in acute setting of thrombosis or long half-life for prophylaxis [3]. Aspirin is the most commonly used antiplatelet drug worldwide in both primary and secondary prevention of CAD. However, the antiplatelet effects of aspirin may vary between individuals. A proportion of patients prescribed with aspirin suffered from recurrent thromboembolic vascular events (“aspirin resistance”) or had increased risk of bleeding [4].

The pharmaceutical industry uses animal polysaccharides on a large scale, especially for treatment of CAD. Heparin is an example of an animal-based medication, widely used as anticoagulant for the treatment and prevention of thrombotic diseases and for maintaining blood fluidity in extracorporeal devices [5]. Unfortunately, the main complication with heparin as an anticoagulant includes occasional life-threatening bleeding and heparin-induced thrombocytopenia [6]. Medicinal plants can serve as a better source for new medication and their potential is now becoming a topic of interest for researchers all over the world. WHO has recommended medicinal plants to be used more effectively in the healthcare system [2].

Three plants, namely, the* Berberis vulgaris *L.,* Teucrium polium *L., and* Orthosiphon stamineus *Benth are herbs that have been widely used in daily diets or medicinal purposes particularly in Asia. These plants are traditionally used for diabetes, hypertension, and kidney stones [7–9]. The promising properties including anticoagulation and antiplatelet from* B. vulgaris, T. polium, and O. stamineus* were studied to determine their potential antiatherothrombotic properties. Polysaccharides isolated from higher plants do not contain sulphate groups and their anticoagulant activity is due to the presence of hexuronic acids residues, like GlcA or GalA, and its derivatives [10].

*B. vulgaris* is also known as “barberry”, which is a thorny shrub with yellow flowers and small red fruits. It is native to Europe and Asia and can be found growing in the wild from Canada to Pennsylvania. In cardiovascular medicine,* B. vulgaris* can be used as an antihypertensive and vasodilator agent [8]. Its active constituents also showed antiarrhythmic, anticholinergic, and cardioprotective effects from ischemia/reperfusion injury [11].* Teucrium polium or Teucrium capitatum L. *is locally known as calpoureh or felty germander. It is mostly found in the Mediterranean region and Middle East. It is a subshrub and classified in the Lamiaceae family (Danihelka, Chrtek Jr and Kaplan, 2012). This medicinal plant is also found abundantly in Southwestern Asia, Europe, and North Africa. In cardiovascular pathology, particularly in the management of CAD risk factors,* T. polium *showed dose-dependent hypotensive, antidiabetic, antioxidant, and anti-inflammatory effects in animal studies. Both* in vivo* and in vitro studies showed apparent antihypertensive effect mediated via cholinergic receptors (Niazmand et al., 2011).* O. stamineus* or* Orthosiphon aristatus* (family: Lamiaceae) is a medicinal plant found in Asian and European countries and is one of the most sought-after medicinal plants, particularly in southeast Asia. OS is also known as “Misai Kucing” and the leaves are commonly consumed as Java Tea. Studies have shown the medical benefits of* O. stamineus *and its possible treatment usage in cardiovascular risk factor management. A study by Sriplang et al. [12] on diabetic rats treated with OS plant showed antidiabetic effects in a dose-dependent manner comparable to glibenclamide (commercial antidiabetic medicine). Besides that, the plasma high-density lipoprotein (HDL) concentration increased significantly in concomitant with decreasing triglyceride concentration, which suggested potential antihyperlipidemia on the same study. The property of antihypertensive of* O. stamineus* extract was noted to decrease blood pressure in hypertensive patients.

All three plants were studied for various cardiovascular effects that are essential in CAD management. These include antihyperlipidemia, cardiotonic and antiarrhythmic effects for* B. vulgaris* [11], anti-inflammatory effect for* T. polium *[13], and antihyperlipidemia effects for* O. stamineus* [12]. Therefore, this study embarks on the determination of potential antiatherothrombotic properties of the plant.

This research embarked to determine the anticoagulation and antiplatelet effects of* B. vulgaris, T. polium, and O. stamineus *on atherothrombotic diseases as an herbal treatment. In comparison to current synthetic medicine, herbal therapy showed lower toxicity, being more readily available and having better compatibility. Thus, the usage of complementary and alternative medicine is more favourable in patients with CAD [14]. Therefore, this study investigated the application of* B. vulgaris, T. polium, and O. stamineus * in vitro.

## 2. **Materials and Methods**

### 2.1. The Source of Plants

Three plants were used in this study, namely,* B. vulgaris *(BV)*, T. polium *(TP), and* O. stamineus *(OS). BV fruits and TP flowers were purchased and imported from certified herbal marketing company in Iran. OS leaves were obtained from Taman Pertanian Universiti, Universiti Putra Malaysia. The voucher specimens were identified by Dr. Mohd Firdaus Ismail of Institute of Biosciences, UPM. The herbarium of the plants was deposited at the Herbarium Biodiversity Unit, Institute of Biosciences, UPM, under reference number SK3207/17 for OS, SK3208/17 for BV and SK3209/17 for TP. The plants were washed with distilled water, dried in an oven at 60°C for three consecutive days, weighed, and stored for further usage.

### 2.2. Preparation of Plants Crude Polysaccharide Extracts (BVPE, TPPE, and OSPE)

The extractions were prepared according to the method described by Yoon et al. [10] with slight modification. Firstly, the dried powder (20g) was suspended in 1 L of absolute methanol and refluxed for 2 hours at 76°C. The suspension was filtered and the residues were suspended in 1 L of 0.1M NaOH and later refluxed for 2 hours at 76°C. The process then continued with centrifugation and neutralization with 1M HCl and then concentrated in a rotary evaporator under reduced pressure. The supernatant was filtered and precipitated with four volumes of absolute methanol and stored at 4°C for 24 hours. Then, the precipitate was filtered with Whatman paper grade number 2 (Whatman House, United Kingdom) and the filtrate was precipitated under 80% ethanol and dried at room temperature overnight. The extract was dissolved in 10 mL distilled water and dialysed for 48 hours under running water. The nondialysed portion was centrifuged to remove insoluble material and the supernatant was lyophilised and stored at -20°C until further analysis.

### 2.3. Preparation of Plants Crude Methanol Extract (BVME, TPME, and OSME)

The methanol extraction was performed according to the method described by Saputri and Jantan [15]. The dried BV fruits, OS leaves, and TP flowers were grinded separately into powder form and 100 g of each plants were macerated in 1 L of absolute methanol at a ratio of 1:10 (w/v) and stirred at 250 rpm in an orbital shaker for 1 hour at room temperature. The crude extracts were then filtered through Whatman filter paper No. 1 (Whatman House, United Kingdom) to separate the residue. The remaining residue was re-extracted twice, and the two extracts were combined. The residual solvent of methanol extract was evaporated under vacuum at reduced pressure and 40°C using a rotary evaporator (Eyela, USA). These crude extracts were then lyophilised and stored at -20°C until further analysis.

### 2.4. Preparation of Plants Crude Aqueous Extract (BVAE, TPAE, and OSAE)

The preparation for aqueous extract followed the common decoction method with slight modification [16]. The dried powders of BV, TP, and OS were put into 10 L beaker separately. For each 100 g of dried powder, 4000 mL of distilled water was added. Then the mixtures were heated up to 70°C to reduce the water content to 1000 mL through evaporation. Subsequently, the residues were filtered and the crude extracts were subjected to lyophilisation and stored at -20°C until further usage.

### 2.5. In Vitro Study of Antiatherothrombotic Effect of BV, OS, and TP

The in vitro study consists of obtaining human blood and incubating the blood sample with different plants extracts to identify for antiplatelet and anticoagulation properties. Plant extract that demonstrated the most optimum antiplatelet and anticoagulation properties was selected to proceed for in vivo study. Three potential medicinal plants (BV, TP, and OS) were chosen in the study and three methods of extraction (aqueous, methanol, and polysaccharide crude extraction) were prepared to screen for the optimum effect of antiplatelet and anticoagulation. Aspirin was used in this experiment as positive control. 1 mg of aspirin was diluted with normal saline into 25.5 *μ*g/mL [17].

### 2.6. The Subjects Studied

The study involved human blood samples to assess anticoagulation and antiplatelet effects of BV, TP, and OS. The study was conducted in accordance with approval by Human Ethic Committee, Universiti Putra Malaysia (UPM) (Approval Number for Human Ethic: UPM/FPSK/100-9/2-JKEUPM (JAM_Feb (13) 03). The in vitro study was conducted in Haematology Department, Faculty of Medicine and Health Sciences, UPM, and Pusat Teknologi Penyelidikan Makanan, MARDI, Serdang. Thirty volunteers were recruited [18]. The volunteers were chosen with the following criteria:

- The inclusion criteria include not suffering from any cardiovascular disease (hypertension, congestive heart failure, and coagulation disorder such as haemophilia A or B) or diabetes, no recent usage of nonsteroidal anti-inflammatory drugs, being nonobese or nonsmokers and free from dyslipidemic disorders and not taking any food within the last eight hours.
- The exclusion criteria include having abnormal platelet counts (less than 200 000 mg/dl) and a history of taking any medication or supplement within fourteen days.

### 2.7. Preparation of Blood Sample

The volunteers were required to fast overnight and the blood was sampled in the morning. Whole blood (20 mL) was withdrawn from volunteers using syringe and needle (21 gauge) and was then put directly into two separate 3.2% anticoagulant trisodium citrate at 9:1 ratio blood tube, with 10 mL each [19]. The blood and the trisodium citrate were thoroughly mixed by inverting the blood tubes several times to prevent blood clotting.

The sample preparation for coagulation test was prepared according to the procedure described by Uprichard, Manning, and Laffan [20]. The blood sample was centrifuged at 1500 g for 10 minutes to obtain the platelet-poor-plasma. The obtained plasma samples from each individual were poured separately in plain tubes using automatic pipette and stored at room temperature for immediate usage within three hours or stored for six months in -80°C.

The blood sample preparation for platelet aggregation test was prepared according to the procedure described by Jantan et al. [17]. All blood samples were tested within three hours of blood collection.

### 2.8. Determination of Total Phenolic Content

Total phenolic contents were evaluated using Folin-Ciocalteu's phenol reagent (Adedapo et al., 2009). The Folin-Ciocalteu reagent was prepared by diluting in water at 1:9 v/v. 5 ml of the plant extract mixed with 5 ml Folin-Ciocalteu reagent. After 5 minutes, 4 ml of 7% Na_2_CO_3_ solution was added and thoroughly mixed with vortex mixer for 5 sec and allowed to stand for 30 min at 40°C for colour development. The absorbance was measured at 765 nm using the Shimadzu UV visible spectrophotometer (UV-1650 PC, Japan). All experiments were conducted 3 times and readings were obtained in triplicate. Samples of plant extracts were evaluated at a final concentration of 0.1 mg/ml. Total phenolic content was expressed as mg/ml tannic acid equivalent using the following equation based on the calibration curve: *γ*=0.608*χ*+0.5057, R^2^= 0.9365, where *γ* was the absorbance and *χ* was the concentration.

### 2.9. Coagulation Test

The anticoagulant activity was determined with prothrombin time (PT) and activated partial thromboplastin time (APTT) following the standard method described by [21]. 250 *μ*L of human platelet-poor-plasma was incubated in a reaction vial with 100 *μ*L of different concentrations of BVME, BVPE, BVAE, TPME, TPPE, TPME, OSME, OSPE, and OSAE (50, 25, and 12.5 mg/mL, respectively) for 7 minutes at room temperature before being subjected to PT and APTT tests.

Neoplastin Cl Plus reagent and STA-PTT automated reagent, Diagnostica Stago (Asnieres-sur-Seine, France), were reconstituted and subjected to PT and APTT assays, together with 0.025M calcium chloride. The PT and APTT assays were analysed using STA Compact coagulation analyser (Diagnostica Stago, France) according to the manufacturer's protocol. The time taken for clot formation was detected using the analyser and compared with the PT and APTT from control (without extracts) as the negative control and plasma mixed with heparin sodium salt (140 IU/mg) (Sigma-Aldrich, USA) as the positive control. All experiments were carried out in triplicate.

### 2.10. Platelet Aggregation Test

Whole-blood platelet aggregation study was performed according to method described by Cardinal and Flower [22] with slight modification. The test was done in Pusat Penyelidikan Teknologi Makanan, MARDI, Selangor. The blood sample was diluted with physiological saline in the ratio of 1:1. The plant extracts were dissolved in saline to obtain serial concentrations of 50, 25, and 12.5 mg/ml, respectively. 5 *μ*L of the plant extract was added to a cuvette containing diluted whole blood and the mixture was incubated at 37°C for four minutes prior to addition of agonist. The agonist used was arachidonic acid (AA) (0.5 mM), collagen (2 *μ*g/mL), or (adenosine diphosphate) ADP (10*μ*M) to fasten the aggregation process. The volume needed for the reaction was 1 mL of the total mixture. The platelet aggregation was measured with whole-blood Lumi-Aggregometer (Chrono-Log Corporation, USA) using an electrical impedance method described by [23]. The mean platelet aggregation in whole blood was identified from the variable in impedance over six minutes after the addition of the agonist, with comparison to that of control group impedance. A mixture containing diluted whole blood was used as control. Aspirin (KCK Pharmaceutical Industries, Malaysia) was used as the positive control. The aggregation was expressed in ohm (Ω) and the readings were obtained after the addition of agonist into the mixture for 6 minutes. Each assay was performed in triplicate.

The calculation for inhibition of platelet aggregation was calculated using following formula [15]:

(1) Inhibition  Percentage  %=1−Sample  AggregationControl  Aggregation×100%

### 2.11. Statistical Analysis

The data were presented as mean ± standard error of mean (SEM). Statistical analysis was carried out by using one-way ANOVA followed by LSD post hoc test. The value was considered significant when* p* value<0.05.

## 3. **Results**

### 3.1. Total Phenolic Compound

*B. vulgaris* aqueous extract has significant amount of phenolic content with mean value of 7.1 mg/mL, compared to* T. polium *and* O. stamineus *with mean values of 2.6 mg/mL and 3.8 mg/mL, respectively (*p*<0.05).

### 3.2. Anticoagulation Effect (PT and APTT) of BV, TP, and OS Using Aqueous and Polysaccharide Crude Extracts

The anticoagulant activity of polysaccharide and aqueous extracts of BV, TP, and OS, respectively, was determined using PT and APTT assay. A one-way analysis of variance (ANOVA) was performed to compare the effect of PT and APTT between groups of extracts followed by LSD post hoc test.

**Figure 1:**
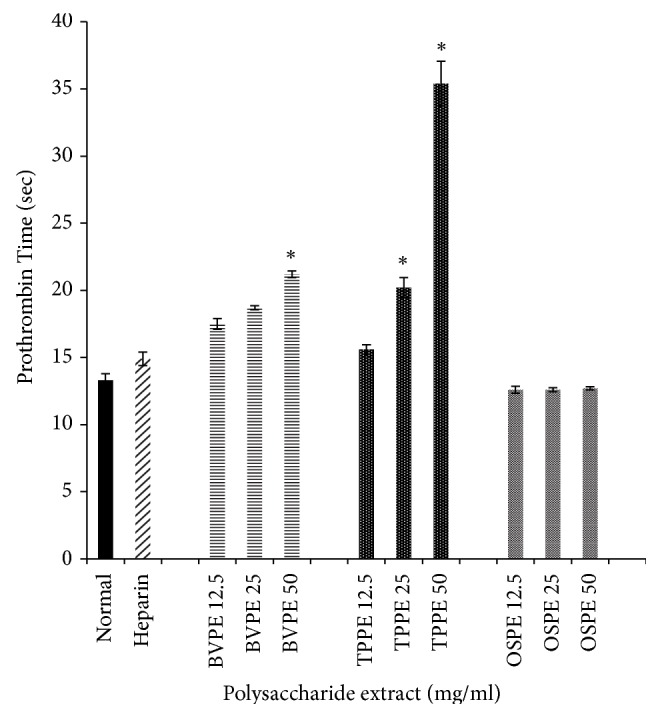
*Effects of BVPE, TPPE, and OSPE on prothrombin time (seconds) of human plasma*. TPPE and BVPE showed prolonged PT in concentration-dependent manner, compared to heparin and normal group. Bars with asterisk showed significant difference compared to normal group at* p*<0.05. BVPE: polysaccharide extract of* B. vulgaris*, TPPE: polysaccharide extract of* T. polium*, OSPE: polysaccharide extract of* Orthosiphon stamineus *Benth.

The baseline PT values of healthy respondents varied between 12.7 and 13.6 seconds. The anticoagulation effects of polysaccharide extracts of BV, TP, and OS on PT are as shown in Figure 1. Both BVPE and TPPE prolonged PT compared to baseline (normal) in a concentration-dependent manner. The PT of all concentration of OSPE groups showed no significant difference to normal group (*p*<0.05). BVPE 50, BVPE 25, BVPE 12.5, TPPE 50, and TPPE 25, respectively, showed significant prolonged PT compared to normal group (*p*<0.05). The highest PT was observed in TPPE 50 group followed by BVPE 50 group.

**Figure 2:**
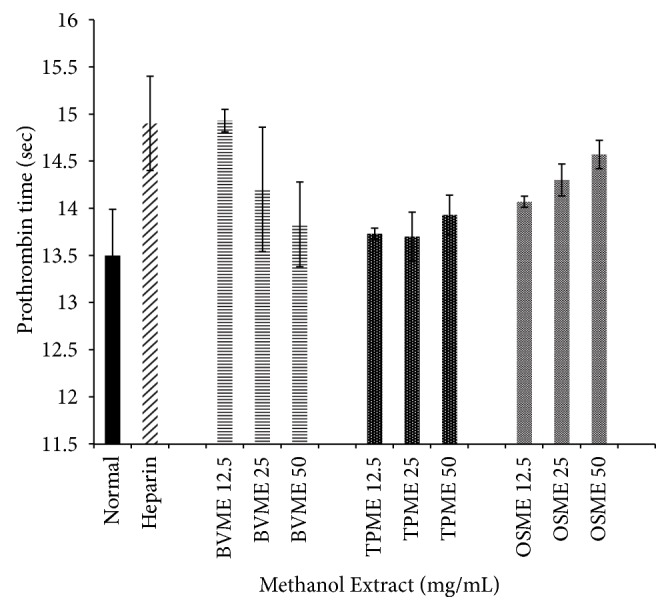
*Effects of BVME, TPME, and OSME on prothrombin time (seconds) of human plasma*. No significant difference was observed compared to the normal group. BVME: methanol extract of* B. vulgaris*, TPME: methanol extract of* T. polium*, OSME: methanol extract of* Orthosiphon stamineus*.

Figure 2 showed the anticoagulation effect of methanol extract of BV, TP, and OS at three different concentrations on PT. No significant difference was observed in BVME, TPME, and OSME with comparison to the normal group (*p*>0.05).

**Figure 3:**
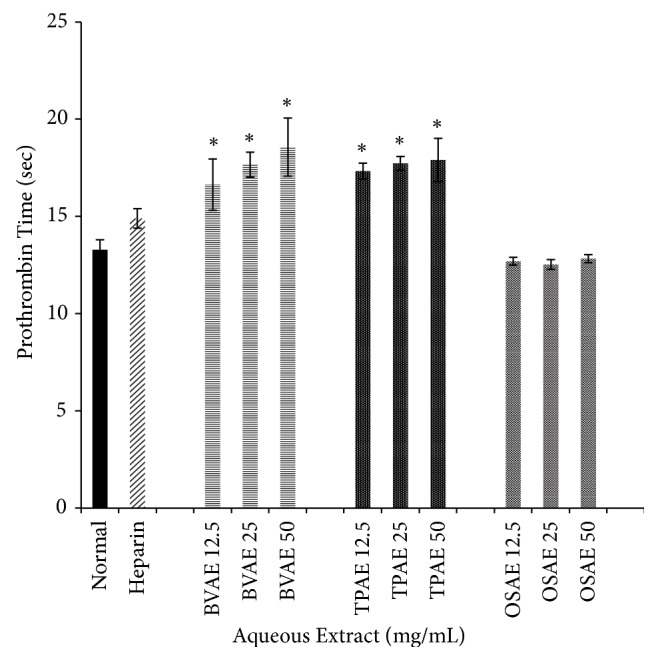
*Effects of BVAE, TPAE, and OSAE on prothrombin time (seconds) of human plasma*. TPAE and BVAE showed prolonged PT in concentration-dependent manner, compared to normal and heparin group. Bars with asterisk showed significant difference compared to normal at* p*<0.05. BVAE: aqueous extract of* B. vulgaris*, TPAE: aqueous extract of* T. polium*, OSAE: aqueous extract of* O. stamineus*.

The anticoagulation effects of aqueous extract of BV, TP, and OS on PT assays were as shown in Figure 3. Both BVAE and TPAE showed dose-dependent anticoagulation properties. The highest PT was observed in BVAE 50 group followed by TPAE group. Prolongation of PT values for BVAE and TPAE in all concentration were significant (*p*<0.05) in comparison to baseline PT (normal) group. However, no significant prolongation of PT was observed in OSAE in all concentration compared to normal group (*p*<0.05).

**Figure 4:**
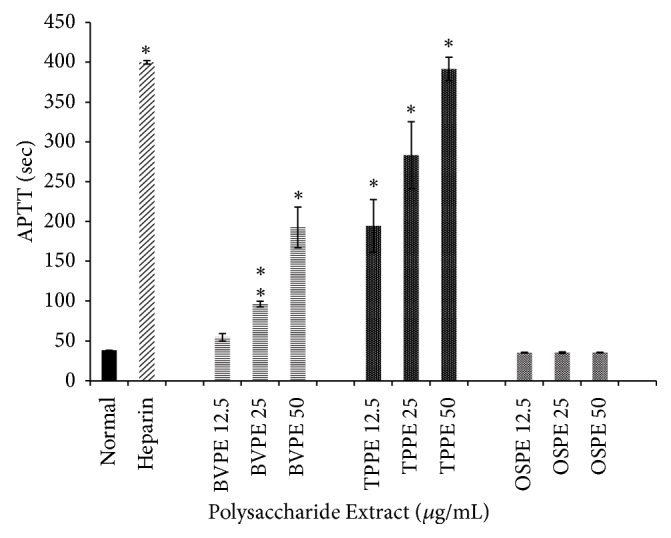
*Effects of BVPE, TPPE, and OSPE on activated partial thromboplastin time in seconds of human plasma*. TPPE and BVPE showed prolonged APTT in concentration-dependent manner, compared to normal group. Heparin (positive control) showed the highest APTT value. Bars with asterisk showed significant difference compared to normal group at* p*<0.05. BVPE: polysaccharide extract of* B. vulgaris*, TPPE: polysaccharide extract of* T. polium*, OSPE: polysaccharide extract of* Orthosiphon stamineus*.

**Figure 5:**
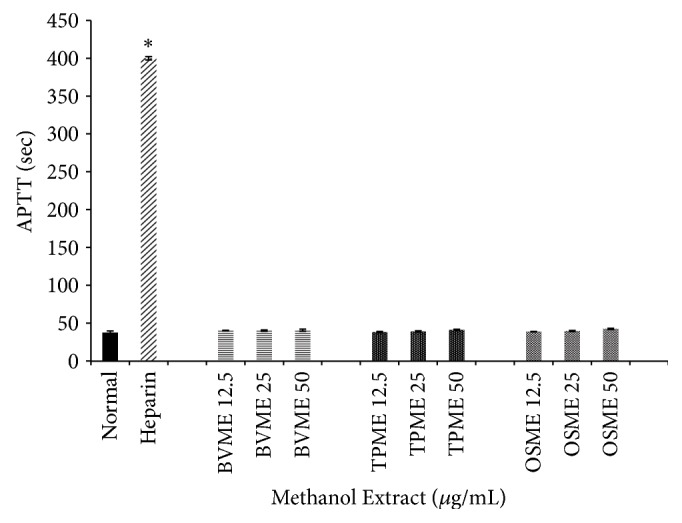
*Effects of BVME, TPME, and OSME on activated partial thromboplastin time (seconds) of human plasma*. No significant difference compared to the normal group. Bars with asterisk showed significant difference compared to normal at p<0.05. BVME: methanol extract of B. vulgaris, TPME: methanol extract of T. polium, OSME: methanol extract of* Orthosiphon stamineus*.

**Figure 6:**
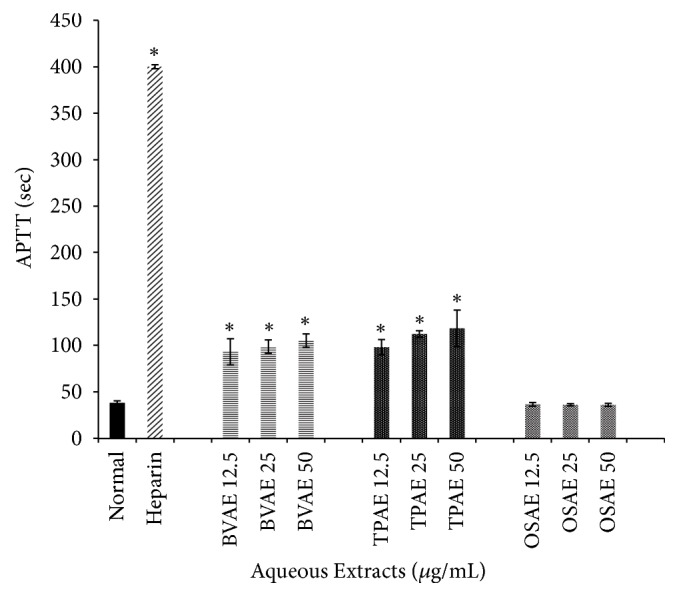
*Effect of BVAE, TPAE, and OSAE on activated partial thromboplastin time in seconds of human plasma*. TPAE and BVAE showed prolonged APTT in concentration-dependent manner, at almost one-third to heparin group. Bars with asterisk showed significant difference compared to normal group at* p*<0.05. BVAE: aqueous extract of* B. vulgaris*, TPAE: aqueous extract of* T. polium*, OSAE: aqueous extract of* Orthosiphon stamineus*.

The anticoagulation effects of BV, TP, and OS extract on APTT assays were as shown in Figures 4, 5, and 6, respectively. The baseline mean for APTT values of healthy human respondents ranged between 36.7 and 40.9 seconds. Heparin (140 IU/mg) was used as positive control with mean value of 400 seconds (maximum detection time for blood coagulation meter).

The anticoagulation effects of polysaccharide extracts of BV, TP, and OS on APTT assays were shown in Figure 4. Both BVPE and TPPE showed concentration-dependent prolongation of APTT. TPPE at all concentrations showed significant prolongation of APTT compared to normal groups (*p*<0.05). At 50 *μ*g/ml concentration of TPPE, maximum APTT was observed with no significant difference compared to heparin (positive control). BVPE at lowest concentration (12.5 mg/ml) showed no significant difference; however, at concentration of 25 and 50 mg/ml, both showed significant difference of APTT compared to normal group (*p*<0.05). All concentrations of OSPE showed no significant prolongation of APTT compared to normal group (*p*>0.05).

Figure 5 shows the anticoagulation effects of methanol extract of all three plants, which include BVME, TPME, and OSME, and these extracts showed no significant prolongation of APTT compared to normal group (*p*>0.05). The anticoagulation effects of BVAE, TPAE, and OSAE were as shown in Figure 6. Both BVAE and TPAE in all concentrations showed significant prolongation of APTT compared to normal groups (*p*<0.05). OSAE in all concentrations showed no significant difference compared to normal groups (*p*>0.05).

Overall, extracts from BV and TP showed significant prolongation of PT and APTT. All types of extraction for OS showed no significant prolongation of both PT and APTT.

### 3.3. In Vitro Study of Antiplatelet Effects of BVAE, BVPE, TPAE, and TPPE

For antiplatelet properties, the experiments were limited to two plants with two-extraction methods. Polysaccharide crude extract and aqueous extract of BV and TP were chosen for the platelet function study. OS was excluded for this test due to limited anticoagulation effect.

Table 1 showed the platelet inhibition activity of BVAE, BVPE, TPAE, and TPPE against collagen, AA, and ADP using human whole blood. The concentration-dependent effects were observed in BVAE, BVPE, TPAE, and TPPE, respectively. The percentages of inhibition of collagen-, ADP-, and AA-induced platelet aggregation by aspirin were 76%, 46.7%, and 100%, respectively. In collagen-induced platelet aggregation assays, BVPE (50 mg/ml) showed inhibition of aggregation which is significantly different compared to control (*p*<0.05). In ADP-induced platelet aggregation assays, none showed significant inhibition compared to control (*p*>0.05). BVAE (50 mg/ml) and BVAE (25 mg/ml) showed significant inhibitory effect of aggregation induced by AA with inhibition of 60.9% and 55.5%, respectively (*p*<0.05). TPAE showed the lowest inhibitory effect of platelet aggregation induced by ADP (11.1%).

## 4. ** Discussion**

### 4.1. Different Methods of Plants Extraction

The extraction of medicinal plant involves the usage of various solvents based on respective ability to extract bioactive compounds of different solubility and polarities. Two plant extraction methods were performed in this study, namely, the aqueous and polysaccharide crude extraction. Both aqueous and methanol extractions were commonly used in medicinal plants investigation as they are polar in nature, and polysaccharide crude extracts were commonly used in atherothrombotic studies [24, 25]. The aqueous extraction product mainly contains metals, ions, high hydrophilic compounds, water-soluble enzymes, glycoproteins, peptides, amino acids, nucleotides, sugar, and polysaccharides, while alcohol extraction product is mainly composed of very polar, neutral, basic, and acidic compounds, amino acids, nucleotides, sugar, polysaccharides, and natural oil [26]. In general, hot aqueous extract is commonly used for herbal remedy and alcoholic extract is more common for chemical analysis and bioassay in laboratory setting.

In this study, aqueous extracts of BV and TP exhibited more potent anticoagulation properties for all parameters tested in in vitro study compared to methanol extract. This supports the exaggerate claims that organic solvent is more effective for extraction, thus overlooking the potential use of water [27]. These results also highlighted the biochemical nature of active compounds in BV fruits and TP leaves that might contribute to anticoagulation properties. Furthermore, the usage of hot water in aqueous extraction method may improve the extraction of compounds responsible for the anticoagulation activity; presumably, the extracted active compounds are thermally labile.

The present study showed that aqueous extract of BV demonstrated more potent antiplatelet effect compared to TP and OS. A study by Saputri and Jantan, 2011 showed the positive correlation between antiplatelet effect and total phenolic compounds in* Garcinia* species. Similar findings by Movahedi, 2014, also demonstrated the optimal antiplatelet effect in correlation to total phenolic compound of BV. All three plants showed different total phenolic compound content with respect to different types of extraction method [28, 29]. Phenolic compounds are important plant metabolites that are commonly present in plants and well known for their antioxidant properties and free radical-scavenging abilities. Several studies also reported the strong correlation between phenolic compound with antioxidant and anti-inflammatory effect [15, 30, 31].

### 4.2. Anticoagulation Effect of BV, TP, and OS Plants Crude Extracts

The assessment on three medicinal plants with three extraction methods was done using STA compact coagulation analyser. The incubation of human platelet-poor-plasma with plant crude extracts resulted in changes of the coagulation properties. The anticoagulation properties of human plasma for aqueous extracts, polysaccharide crude extracts, and methanol extracts were tested using APTT and PT assays, respectively.

Both aqueous and polysaccharide crude extracts of* B. vulgaris* and* T. polium *showed significant prolongation of APTT and PT compared to baseline value. Polysaccharide crude extract of both plants showed stronger anticoagulant activities compared to the baseline coagulation time in concentration-dependent manner.

This study demonstrated that polysaccharide crude extract of both plants appeared to have anticoagulant properties. Prolonged PT suggested the presence of clotting inhibitor in the extracts which interferes with the integrity of coagulation proteins, especially factor VII in the extrinsic coagulation pathway [32]. However, certain condition may lead to false positive result (prolonged PT), which includes the usage of frozen-thawed plasma specimens, by which the activity of coagulation factor V is slightly lower up to 20% from normal [33]. Thus, to avoid this possibility, the normal group PT tests were run together with other plant extracts for each set of tests. As expected, heparin showed slight effect on PT with mean value of 14.9 seconds. The reason being is the APTT test is more sensitive towards detection of antithrombin-dependent mechanism.

As for APTT, polysaccharide crude extracts (TPPE and BVPE) showed the highest activity of anticoagulation followed by aqueous extract for both plants (TPAE and BVAE) in a concentration-dependent manner. In this study, the prolongation of APTT showed that TPPE might have great potential as an anticoagulant and antiplatelet agent. Meanwhile BVPE 50 took almost half of the maximum coagulation time.

According to Mayo Clinic [34], the therapeutic APTT range is approximately between 70 and 120 seconds. All tests on three concentrations of TPAE and BVAE and BVPE 25 extracts showed prolongation time of APTT, which was within the therapeutic APTT range time. This proved that the aqueous extracts of* B. vulgaris* and* T. polium* are safe and effective as anticoagulation agents.

Prolongation of APTT suggested the inhibition of intrinsic and common pathways; meanwhile, the prolongation of PT implied the inhibition of extrinsic coagulation pathway [35]. Polysaccharide crude extract of both* B. vulgaris* and* T. polium *showed significant prolongation of APTT. In comparison to heparin, a well-known sulphated polysaccharide showed the optimum effect of anticoagulant [36]. Heparin catalysed the inhibition of thrombin formation by binding to antithrombin III (heparin cofactor) thus inactivating activated factor X [37]. Both TP and BV polysaccharide extracts showed significant anticoagulation effect compared to aqueous and methanol extracts. The prolongation assay of PT and APTT demonstrated by these polysaccharides may cause the interference of thrombin and antithrombin activity. This might further lead to inhibition of other coagulation factors including factor Xa, factor VIIa, factor Ixa, and factor XIIa that subsequently prolonged the PT and APTT.

The anticoagulant activities of polysaccharide-based plants are well documented in several studies. Khoo et al. [25] showed that polyphenolic-polysaccharide isolated from* M. malabathricum* L. potentially prolonged blood clotting in the intrinsic pathway as anticoagulant agent. Yoon et al. [10] stated that the anticoagulant activity of* Porana volubilis* was contributed by acidic polysaccharides and polyphenolic compounds.

### 4.3. Antiplatelet Effects of BV and TP Crude Extracts

BV and TP extracts were subjected for further platelet function test, as OS showed no effect on coagulation and no possible antiplatelet activities. Study done by Saputri and Jantan [15] showed poor inhibition of platelet aggregation by* O. stamineus* when induced by AA, ADP, and collagen. BVAE, BVPE, TPAE, and TPPE inhibited platelet aggregation in a dose-dependent manner, by which the percentage of inhibition increased as the concentration of the extract increased.

In this study, BVAE 50 showed the highest AA-induced platelet inhibition. The significant inhibition reaction on AA-induced platelet aggregation is similar to aspirin, as positive control drug showed 100% inhibition in platelet aggregation study. Aspirin, on the other hand, showed lower inhibition pattern against ADP and collagen-induced platelet aggregation. BVPE and BVAE showed lower inhibition pattern against collagen and ADP-induced platelet aggregation compared to AA-induced platelet aggregation. These findings on different percentage of platelet inhibition against different agonists on similar extract concentration were supported by previous studies on aspirin,* Garcinia eugenifolia* and* Camellia sinensis* [17, 38].

Total phenolic compound was shown to have positive correlation with antiplatelet effect [39]. A study by (Movahedi, 2014) showed that* B. vulgaris* significantly contains higher total phenolic compound compared to* T. polium*. This supported the finding in this study which showed BV had higher antiplatelet properties than TP [40]. Conversely, BV aqueous extract showed stronger inhibition pattern compared to BV polysaccharide extract. Polysaccharide extract of TP showed stronger inhibition pattern compared to aqueous extract of TP. The incongruent effects of similar extraction from different plants might provide the hypothesis on other unidentified factors that might have caused the antiplatelet effect in both plants [41, 42].

Previous studies have shown berberine as one of the main active compounds of* B. vulgaris *[43, 44]. Berberine demonstrated antiplatelet aggregation potential through inhibition of TXA2 induced by ADP, AA, and collagen [45]. According to the study, berberine significantly inhibited rabbit platelet aggregation induced by ADP, AA, and collagen. The study also showed that berberine significantly inhibited synthesis of thromboxane A2 (TXA2) in rabbit platelet. Collagen-induced TXA2 synthesis was also most potently inhibited at 50 mg/kg berberine with mean value of 80.6% inhibition. However, in this study, the administration of BVAE has showed significantly potent antiplatelet effect on both AA-induced and collagen-induced platelet aggregation with respect to that of specific berberine compound. Thus, the mechanisms related to antiplatelet effect of BVAE might be due to AA metabolism of TXA2 in platelet and endothelial cells at cyclooxygenase of AA cascade [45].

## 5. Conclusion

The evidences from this study contribute to the new findings towards potential antithrombotic agent from* B. vulgaris* and* T. polium*. However, further studies can to be carried out to isolate the bioactive compounds as well as* in vivo* studies with maximum inhibitory activity of coagulation and platelet aggregation. These medicinal plants may provide future contribution towards alternative treatment for cardiovascular disease via anticoagulation and antiplatelet effects.

## Figures and Tables

**Table 1:** Inhibition of platelet aggregation (%) in human whole blood with BVPE, BVAE, TPPE, and TPAE using platelet agonists of collagen (2 *μ*g/ml), ADP (10 *μ*M), and AA (0.5 *μ*M).

Sample	Concentration	Inhibition of Platelet Aggregation (%)		
	(*μ*g/ml)	Collagen	ADP	AA
BVPE	12.5	40.4 ± 3.4	23.8 ± 3.9	49.6 ± 3.4
	25	45.8 ± 2.8	26.7 ± 2.6	52.0 ± 3.1
	50	53.7 ± 6.9*∗*	35.9 ± 3.1	52.6 ± 2.6

BVAE	12.5	39.6 ± 3.1	34.1 ± 3.7	49.3 ± 2.8
	25	44.4 ± 3.6	36.3 ± 3.9	55.5 ± 3.5*∗*
	50	47.1 ± 3.6	48.9 ± 2.8	60.9 ± 2.9*∗*

TPPE	12.5	31.6 ± 3.2	26.5 ± 4.3	36.2 ± 2.2
	25	39.2 ± 4.6	35.7 ± 4.6	43.7 ± 3.8
	50	42.2 ± 4.4	48.4 ± 4.1	54.3 ± 4.5

TPAE	12.5	25.8 ± 4.1	11.1 ± 3.9	42.0 ± 4.5
	25	32.9 ± 4.1	26.7 ± 3.5	44.1 ± 3.6
	50	36.3 ± 4.4	44.4 ± 3.9	45.6 ± 3.3

Aspirin	30	76 ± 3.3*∗*	46.7 ± 4.2	100*∗*

Aspirin was used as a positive control. Results were expressed as mean ± SEM. BVAE showed highest inhibition in AA-induced platelet aggregation. *∗*<0.05 as compared with respective control.

## Data Availability

The data used to support the findings of this study are available from the corresponding author upon request.
